# *In Silico* Evidence for Gluconeogenesis from Fatty Acids in Humans

**DOI:** 10.1371/journal.pcbi.1002116

**Published:** 2011-07-21

**Authors:** Christoph Kaleta, Luís F. de Figueiredo, Sarah Werner, Reinhard Guthke, Michael Ristow, Stefan Schuster

**Affiliations:** 1 Department of Bioinformatics, School of Biology and Pharmaceutics, Friedrich Schiller University of Jena, Jena, Germany; 2 Systems Biology/Bioinformatics Group, Leibniz Institute for Natural Product Research and Infection Biology – Hans Knöll Institute, Jena, Germany; 3 Department of Human Nutrition, Institute of Nutrition, University of Jena, Jena, Germany; 4 Department of Clinical Nutrition, German Institute of Human Nutrition, Potsdam-Rehbrücke, Nuthetal, Germany; Stanford University, United States of America

## Abstract

The question whether fatty acids can be converted into glucose in humans has a long standing tradition in biochemistry, and the expected answer is “No”. Using recent advances in Systems Biology in the form of large-scale metabolic reconstructions, we reassessed this question by performing a global investigation of a genome-scale human metabolic network, which had been reconstructed on the basis of experimental results. By elementary flux pattern analysis, we found numerous pathways on which gluconeogenesis from fatty acids is feasible in humans. On these pathways, four moles of acetyl-CoA are converted into one mole of glucose and two moles of CO_2_. Analyzing the detected pathways in detail we found that their energetic requirements potentially limit their capacity. This study has many other biochemical implications: effect of starvation, sports physiology, practically carbohydrate-free diets of inuit, as well as survival of hibernating animals and embryos of egg-laying animals. Moreover, the energetic loss associated to the usage of gluconeogenesis from fatty acids can help explain the efficiency of carbohydrate reduced and ketogenic diets such as the Atkins diet.

## Author Summary

That sugar can be converted into fatty acids in humans is a well-known fact. The question whether the reverse direction, i.e., gluconeogenesis from fatty acids, is also feasible has been a topic of intense debate since the end of the 19^th^ century. With the discovery of the glyoxylate shunt that allows this conversion in some bacteria, plants, fungi and nematodes it has been considered infeasible in humans since the corresponding enzymes could not be detected. However, by this finding only a single route for gluconeogenesis from fatty acids has been ruled out. To address the question whether there might exist alternative routes in humans we searched for gluconeogenic routes from fatty acids in a metabolic network comprising all reactions known to take place in humans. Thus, we were able to identify several pathways showing that this conversion is indeed feasible. Analyzing evidence concerning the detected pathways lends support to their importance during times of starvation, fasting, carbohydrate reduced and ketogenic diets and other situations in which the nutrition is low on carbohydrates. Moreover, the energetic investment required for this pathway can help to explain the particular efficiency of carbohydrate reduced and ketogenic diets such as the Atkins diet.

## Introduction

**Figure 1:**
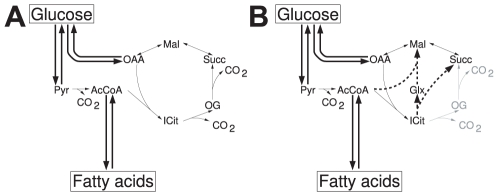
Classical scheme of the interconversion between glucose and fatty acids in humans. **A** While the conversion of glucose into fatty acids is possible, the product of β-oxidation of even-chain fatty acids, acetyl-CoA, can only enter the TCA cycle by reaction with oxaloacetate to citrate. However, in order to replenish the oxaloacetate consumed in this reaction, the TCA cycle has to be used resulting in the release of two carbon atoms in form of carbon dioxide. Hence, while two carbon atoms enter the TCA cycle in form of acetyl-CoA two others are lost and a net production of oxaloacetate which would be required for gluconeogenesis is not possible. **B** In some organisms the carbon releasing steps of the TCA cycle can be bypassed using the glyoxylate shunt (thick dotted reactions). In consequence, one mole of oxaloacetate can be produced from two mole of acetyl-CoA, allowing for gluconeogenesis from fatty acids. A list of abbreviations can be found in Table S1.

It is well known that excess sugar in the human diet can be converted both into glycerol and fatty acids and, thus, into lipids such as triglycerides. A related question biochemistry students are often asked in their exams is whether the reverse route is also feasible, that is, whether the human body can convert fatty acids back into glucose. As for even-chain fatty acids, the expected answer is “No” (odd-chain fatty acids practically do not occur in mammals). This summarizes the result of a debate that dates back to the late 19th century. However, it was not until the 1950s that such a conversion could be monitored using ^14^C labeled fatty acids [1]. It was found that part of the label arrives at glucose, proving that there is a connected route from acetyl-CoA to glucose. However, as shown mathematically [1], [2], there cannot be any sustained conversion at steady state along the tricarboxylic acid (TCA) cycle due to stoichiometric constraints. In particular, oxaloacetate would not be balanced (Fig. 1A). A possible route that does allow this conversion in some prokaryotes [3], [4], plants [5], fungi [6] and nematodes [7] is the glyoxylate shunt. It produces an additional oxaloacetate, thus balancing this compound (Fig. 1B). However, the corresponding enzymes have not been found in mammals in spite of controversial speculations [8]. Reports that the glyoxylate shunt would be present in some hibernating animals [9] were not confirmed. In a recent experimental work they have been genetically introduced into mice [10].

In summary, there is a general consent that carbohydrates cannot be produced from even-chain fatty acids in humans although the opposite conversion is feasible. In consequence, this statement can be found throughout prominent biochemistry textbooks [11], [12], [13]. It has even been used as a benchmark criterion for the reconstruction of whole-cell metabolic networks in hepatocytes [14]. An alternative reconstruction of hepatocyte metabolism [15] however, does not use that criterion. This problem is of particular importance with respect to the provision of energy to the brain in situations of drastically reduced carbohydrate uptake. Although the brain can use ketone bodies in these situations, it still needs a certain amount of glucose [16], which has critical implications upon starvation and similar conditions.

**Figure 2:**
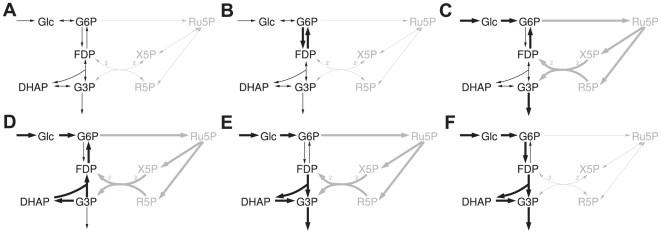
Examples of elementary flux patterns. **A** Schematic network of upper glycolysis and the pentose phosphate pathway with corresponding elementary flux patterns. Reactions of the subsystem are drawn in black. **B–F** Elementary flux patterns of the system. Thick black arrows correspond to the reactions of each flux pattern in the subsystem. Thick gray arrows indicate the reactions used by an elementary mode of the entire system using the reactions of the flux pattern in the subsystem. Individual flux ratios have been omitted for clarity. A list of abbreviations can be found in Table S1.

Recently, we re-investigated the problem in question in a small model of human central metabolism [2]. We used the concept of elementary flux modes [17], which allows one to detect all feasible metabolic pathways in small to medium-scale reaction networks. We were able to corroborate the results of Weinman *et al* [1]. However, only a small part of metabolism rather than the entire human metabolic network was considered. Hence, alternative potential pathways for gluconeogenesis from fatty acids via acetone, such as those proposed in the 1980s [18], [19] could not be detected. In order to check for the existence of such pathways in a holistic approach, we used the most detailed reconstruction of the human metabolic network [20], which had been reconstructed on the basis of experimental results. Moreover, we employ the concept of elementary flux patterns, which allows an analysis in whole-cell metabolic networks similar to elementary mode analysis [21]. An elementary flux pattern corresponds to a set of reactions within a subsystem of a reaction network that is compatible with feasible pathways through the entire system (Fig. 2). Since the entire system is explicitly taken into account, this method allows one to detect all feasible routes on which one compound can be converted into another (for details see *Materials and Methods*). Equipped with this new modeling technique and a genome-scale model of human metabolism, we want to answer the question whether carbohydrates can be produced from fatty acids in humans.

The question whether fatty acids can be converted into carbohydrates has many biochemical implications: effect of starvation (cf. Owen *et al.* [16]), sports physiology [22], the traditional diet of inuit, which is practically carbohydrate-free [23] and high in fish and seal oil. In earlier times, low-carbohydrate diets were also typical for native Americans hunting in the plains. Also the survival of hibernating animals [24] and of embryos of egg-laying animals is crucially dependent on gluconeogenesis [25]. Moreover, the efficiency of carbohydrate reduced and ketogenic diets such as Atkins diet is worth being studied from this viewpoint. In particular, we will introduce two quantities and compute them for fatty acids and various other storage compounds: gluconeogenic energy efficiency and glucose storage efficiency. The former measure quantifies how much energy in form of ATP is regained if a compound is used for gluconeogenesis and is subsequently catabolized. The latter measure quantifies how much glucose is regained if a compound is produced from glucose and subsequently converted back into glucose.

## Results

### Determining pathways for gluconeogenesis from fatty acids

#### Step 1: Elucidating an initial pathway

In a first step, we checked whether there exists a pathway producing glucose 6-phosphate (G6P) from acetyl-CoA in the network of human metabolism described in *Materials and Methods*. This question can be answered by computing the elementary flux pattern of a subsystem just encompassing the inflow reaction of acetyl-CoA and the outflow reaction of G6P. Indeed, we found two elementary flux patterns: one of them contains only the inflow of acetyl-CoA and the other additionally contains the outflow of G6P. The second elementary flux pattern is of special importance since it is a proof of the existence of a pathway in humans that produces G6P from acetyl-CoA, that is, there exists a gluconeogenic route from fatty acids in humans.

**Figure 3:**
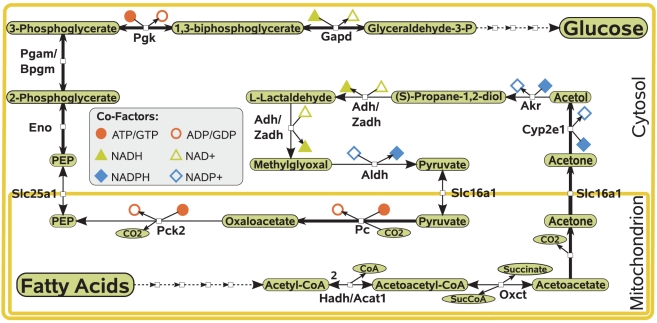
Pathway for the conversion of fatty acids into glucose. Essential reactions of the pathway are drawn with bold arrows. The presented pathway corresponds to the energetically most efficient pathway for the conversion of fatty acids into glucose (pathway 7 in Text S1). A similarly efficient pathway, pathway 14, uses almost the same route but converts acetol directly into methylglyoxal by reduction with NADPH. A list of abbreviations can be found in Table S1.

In order to analyze this route in more detail and to determine whether alternative pathways exist, we determined the global pathway connecting acetyl-CoA and G6P using a procedure described previously [21]. Starting from acetyl-CoA, proceeding via several reactions of ketogenesis and acetone degradation, this pathway finally yields pyruvate which is a common gluconeogenic precursor (Fig. 3). Strikingly, the pathway proceeds via acetone and is similar to the route which has been put forward in [18] and [19].

#### Step 2: Determining essential reactions

Next, we checked which reactions are essential on the above pathway using an algorithm described in Text S1. On the pathway we detected three sequences of essential reactions (Fig. 3): the conversion of mitochondrial acetoacetate into cytosolic acetol, the carboxylation of pyruvate to oxaloacetate in the mitochondrion and cytosolic gluconeogenesis from phosphoenolpyruvate. Since the conversion of acetoacetate into acetol proceeds via acetone, this metabolite is an intermediate in all routes.

#### Step 3: Determining alternative routes

After having identified essential reactions of the gluconeogenic pathway we aimed to elucidate the parts of the pathway on which variable routes could be used. Since these alternative routes have to connect the previously identified sequences of essential reactions we could deduce that they perform one of the following biochemical functions:

1. Conversion of mitochondrial acetyl-CoA into mitochondrial acetoacetate
2. Conversion of cytosolic acetol into mitochondrial pyruvate
3. Conversion of mitochondrial oxaloacetate into cytosolic phosphoenolpyruvate

The latter mainly concerns well-known routes for the transport of oxaloacetate and other intermediates of the mitochondrial TCA cycle into the cytosol. Therefore, we will concentrate on conversions (1) and (2) in the following.

**Figure 4:**
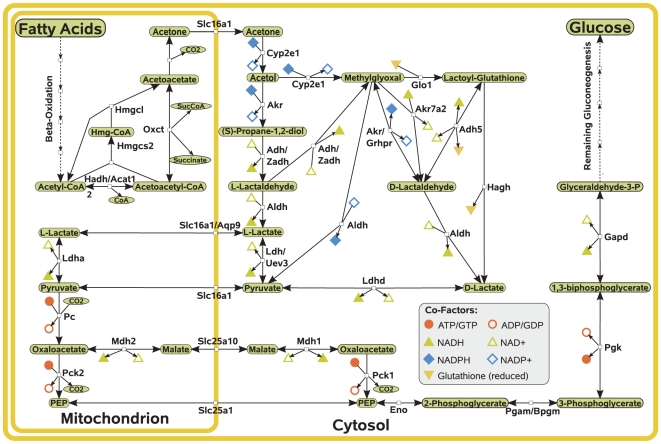
Gluconeogenic pathways from fatty acids. Only mitochondrial pathways for ketogenesis are shown since they represent the most important routes. Aqp9, Akr and one form of Acat2 are noted as irreversible in the model, but are lumped together with reversible reactions in the figure. A list of abbreviations can be found in Table S1. A complete list of pathways in ketogenesis and acetone metabolism is given in Text S1.

Using the procedure described in *Materials and Methods* we obtained two subsystems: one containing alternative pathways in the conversion of acetyl-CoA into acetoacetate and the other containing alternative pathways in acetone degradation. The pathways within these subsystems are depicted, along with two gluconeogenic routes from pyruvate, in Fig. 4.

### Pathways for gluconeogenesis from fatty acids

Overall we detected nine possible pathways for the conversion of acetyl-CoA into mitochondrial acetoacetate (see Text S1). In acetone degradation we identified 58 possible pathways.

In addition to the work of [26] in which acetone metabolism was reviewed, we identified a new intermediate of acetone metabolism, D-lactaldehyde, which is used in 14 out of the 58 pathways. This metabolite can be produced from either methylglyoxal or lactoyl-glutathione, an intermediate in the conversion of methylglyoxal to D-lactate. The conversion of methylglyoxal to D-lactaldehyde is catalyzed by an aldo-keto reductase or by a glyoxylate reductase. Aldo-keto reductase is only weakly expressed in the liver [27]. Thus, this pathway might be of greater importance in other tissues. Another important point is the import of pyruvate into mitochondria. Pyruvate can be either directly imported into the mitochondrion or indirectly via a lactate shuttle [28] (Fig. 4). We will not consider the indirect import since its importance is disputed [29]. Therefore, only 22 pathways for the conversion of acetol into pyruvate remain (Text S1).

One important feature of the described pathways is that many of them involve the reduction of NAD^+^ or oxidation of NADPH. Overall, 1–3 moles of NADPH are oxidized and 0–4 moles of NAD^+^ are reduced during the conversion of one mole of acetone into pyruvate (see Text S1). Special emphasis can be put on the oxidation of methylglyoxal to pyruvate. In this case, one mole of NADP^+^ can be reduced leading to pathways in which the net balance in oxidized NADPH is only one (pathways 7 and 14, depicted in Fig. 3). Pathways not using this reaction have a net balance of at least two moles of NADPH oxidized. As discussed below, these requirements on the reduction potential impose constraints on the utilization of gluconeogenic routes from fatty acids.

### Comparison to other pathway detection techniques

Besides the concept of elementary flux patterns, other techniques to determine pathways in genome-scale metabolic networks exist. Among the most widely used techniques is flux balance analysis [30] that allows one to identify physiologically feasible fluxes within a metabolic network that optimize a certain objective function. While a single pathway for the conversion of fatty acids in gluconeogenesis can be obtained with this method, a systematic identification of all possible pathways is not possible.

Another frequently used tool is elementary flux mode analysis [17] that allows one to enumerate all physiological feasible flux distributions in a metabolic network. However, the number of extreme pathways [31], a subset of the elementary flux modes, is estimated to be 10^29^ for the human genome-scale network [32], which makes the full enumeration of all the elementary flux modes impossible. Nevertheless, recently two approaches to enumerate subsets of elementary flux modes with increasing number of reactions have been developed [33], [34]. Here, we used the *K*-shortest EFM method to compute the 100 shortest elementary flux modes producing cytosolic glucose 6-phosphate from mitochondrial acetyl-CoA. Within these elementary flux modes only nine of the 22 pathways for the conversion of acetone to pyruvate were present (Text S1). Hence, the complete enumeration of all possible pathways for the conversion of acetone to pyruvate on the pathway of gluconeogenesis from fatty acids could not be achieved by this approach.

## Discussion

### Evidence for gluconeogenesis from fatty acids

We performed a global survey of gluconeogenic routes from fatty acids in human metabolism using a genome-scale metabolic model and elementary flux pattern analysis. Even though prominent biochemistry textbook negate the existence of such a route in mammals and humans in particular [11], [12], [13], we were able to confirm several routes that have been proposed earlier [18], [19]. Additionally, we were able to identify 14 new pathways that proceed via a new intermediate in acetone metabolism, D-lactaldehyde. These results further underline the utility of elementary flux pattern analysis in assessing the metabolic capabilities of organisms.

Gluconeogenesis becomes important when the glucose level in the body cannot be sustained by the glycogen store in the liver, which is sufficient for up to one day in humans [16]. Such a situation arises, for instance, during starvation, fasting, prolonged physical exercise, upon carbohydrate-reduced and ketogenic diets like Atkins diet as well as in hibernating animals. In addition to glucogenic amino acids from proteins, the glycerol component of lipids can be converted into glucose while fatty acids serve as principal metabolites to fuel oxidative phosphorylation. In prolonged starvation, not only muscular protein but also proteins essential for the maintenance of principal body functions are broken down to serve for gluconeogenesis [16]. However, there is obviously a limit to such protein degradation.

While the principal fuel of the brain under normal conditions is glucose, in starvation, the brain starts using ketone bodies in addition to reduced consumption of glucose. The increase of the production of ketone bodies leads to rising levels of acetoacetate, which is constantly decarboxylated into acetone. For example, in 21 days fasted humans, 37% of the acetoacetate is converted into acetone [35]. The putative enzyme catalyzing this reaction, acetoacetate decarboxylase, has been characterized in terms of catalytic activity [36] and inhibitors [37], but has not yet been identified [26]. 2–30% of acetone is excreted via the urine and breath [35], while the remainder is metabolized further and could account for up to 11% of gluconeogenesis during starvation [35]. Glucose formation from acetone was indeed suggested as an explanation for the finding that common gluconeogenic precursors alone could not fully account for renal gluconeogenesis [16]. Additional supporting evidences come from the increased activity of cytochrome P450 (Cyp2e1), an enzyme essential in the presented pathways, during starvation [38] and the observation that increased survival time of obese rats during starvation correlates with the activity of acetone metabolism [39]. Moreover, increased levels of methylglyoxal, an intermediate of some of the gluconeogenic routes from fatty acids, have been observed in subjects on the carbohydrate reduced Atkins diet during which ketogenesis is particularly active [40]. Thus, these pathways play an important role in gluconeogenesis also in other situations like the ones mentioned in the Introduction in which ketogenesis is particular active. In hibernating animals, for example, protein may be saved by not using it as the only source for gluconeogenesis. This hypothesis is confirmed by recent results in which it was found that Cyp2e1 and phosphoenolpyruvate carboxykinase (Pck1), both enzymes within the described pathways, are significantly (p = 0.005) upregulated in the liver of hibernating black bears (FoldChange 5.990 and 13.17, respectively) [41]. In a more recent work, also Pck1 (FC = 10.7) and Pc (FC = 2.62) were found to be upregulated in the liver of the animals during hibernation [42].

### Limited capacity of gluconeogenesis from fatty acids

Important aspects related to the presented pathways are energetic requirements that constrain their capacity. Experimental investigation of the gluconeogenic role of acetone showed differences in its utilization between species: the net synthesis of glucose from acetone as observed in murine hepatocytes [43] does not seem to occur in perfused rat liver [44]. Instead, net synthesis from acetone could be demonstrated in either case when also other gluconeogenic substrates were given [43]. This can be explained by the increased requirement for NADPH in the conversion of acetone to pyruvate. Each possible pathway requires the oxidation of at least two moles of NADPH (see Fig. 4). However, there are only two pathways which reduce the net balance in NADPH to-1 moles by the direct conversion of methylglyoxal to pyruvate (pathways 7 and 14, Fig. 3). During gluconeogenesis, cytosolic pathways for the replenishment of NADPH are impaired since they would further deplete gluconeogenic metabolites [45]. In fact, NADPH has to be supplied from mitochondrial sources and its availability represents the rate-limiting factor in acetone metabolism [39], [46].

**Figure 5:**
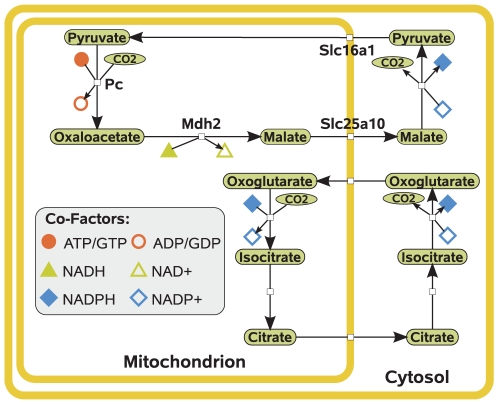
Pathways for cytosolic NADPH production from mitochondrial NADH/NADPH. The upper pathway depicts the transfer of electrons from mitochondrial NADH to cytosolic NADPH. The lower pathway depicts a transfer of electrons from mitochondrial NADPH to cytosolic NADPH. Please note that the second pathway involves the transport of oxoglutarate and citrate over the mitochondrial membrane and necessitates the antiport of L-malate and phosphoenolpyruvate, respectively. A list of enzymes can be found in Table S1.

There are two known pathways of NADPH replenishment from mitochondria [45](Fig. 5). One of them involves the transport of mitochondrial NADPH into the cytosol and the other the transfer of electrons from mitochondrial NADH to cytosolic NADP^+^. The direct transport from the mitochondrion involves a citrate∶oxoglutarate shuttle. Since both metabolites are intermediates of the TCA cycle, they would potentially reduce mitochondrial fatty acid oxidation when used for this shuttle system. The other pathway of NADPH replenishment uses a route via cytosolic malic enzyme, which decarboxylates malate to pyruvate. Both metabolites are intermediates of gluconeogenesis from acetone. Hence, each oxidized NADPH would require an additional cycle in the conversion of pyruvate to malate. Each of these cycles involves the hydrolysis of one ATP to ADP and the oxidation of one NADH. Especially pathways 7 and 14 in the conversion of acetol to pyruvate are of interest since they only require a net consumption of one mole of NADPH per mole of pyruvate produced. Thus, only a single pyruvate-malate cycle would be necessary to balance NADPH consumption. Since there are several possible pathways in acetone metabolism, there might be species differences in the principal pathways which are used for acetone degradation.

Another reason for the limited capacity of the pathways in question is that several of their intermediates, such as methylglyoxal and acetone, are toxic in higher or even moderate concentrations. Thus, the organisms can use these pathways only to a limited extent. A further limiting factor may arise if the decarboxylation of acetoacetate indeed proceeds spontaneously. As it had been observed that Caucasian people can adapt to the diet of inuit within about three weeks [23], the limitation in capacity of these pathways is likely to get less severe over time, probably due to induction of specific enzymes along those pathways.

### Energetic characteristics of gluconeogenesis from fatty acids

**Figure 6:**
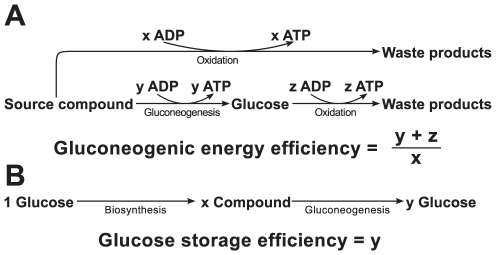
Pathway overview and storage efficiencies of selected compounds. **A** Overview of ATP, NADPH and NADH consumption for gluconeogenesis from acetyl-CoA for all detected pathways (see Text S1). Furthermore, Gibbs free energy changes of all pathways for gluconeogenesis from acetyl-CoA and palmitate are given. **B** Gluconeogenic energy efficiencies and glucose storage efficiencies for selected compounds. Amino acids marked with an asterisk cannot be synthesized from glucose in humans. “Palmitic acid (eff.)” refers to the most energy efficient pathway for gluconeogenesis from fatty acids (pathway 7) and “Palmitic acid (ineff.)” to the most inefficient pathway (pathway 20).

We investigated the energetic requirements of the presented pathways in terms of ATP consumption under the assumption that all other metabolites, including NADPH and NADH need to be balanced (Fig. 6A and Text S1). We found that the presented pathways consume 6–22 moles of ATP for the production of one mole of glucose and two moles of CO_2_ from four moles of acetyl-CoA (requiring a flux of two through the described pathways). The most efficient pathway in terms of ATP consumption proceeds via the oxidation of methylglyoxal to pyruvate, oxidizes two moles of NADPH and reduces two moles of NADH (pathway 7, displayed in Fig. 3). However, since the mitochondrial NADH concentration is already high during ketogenesis [47], pathway 14 might be more favorable because even two moles of cytosolic NADH are reduced on this pathway. Using this pathway the cost for the synthesis of one mole of glucose is increased to 16 moles of ATP.

Moreover, we computed the Gibbs free energy change of the overall pathways to check whether they are thermodynamically feasible (Fig. 6A). We performed these computations for gluconeogenesis starting from acetyl-CoA and palmitate, respectively. Thus, we found that the overall Gibbs free energy change is in the range of −1162 to −1449 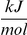 for gluconeogenesis from acetyl-CoA and in the range of −1345 to −1610 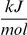 for gluconeogenesis from palmitate. Thus, the presented pathways are all thermodynamically feasible.

### Gluconeogenic energy efficiency and glucose storage efficiency of fatty acids

**Figure 7:**
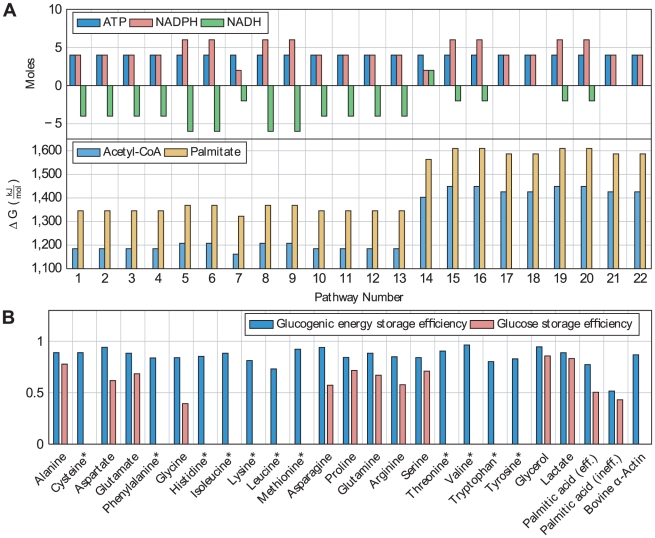
Gluconeogenic energy efficiency and glucose storage efficiency.

In order to analyze the role of the described pathways during prolonged starvation and fasting, we computed for amino acids, fatty acids, lactate and glycerol how much energy in form of ATP is regained in the net balance if they are used for gluconeogenesis and subsequently catabolized. This quantity, termed gluconeogenic energy efficiency, is defined as the ratio of the above mentioned net amount of energy and the energy that would be obtained by catabolizing the substrate directly (Fig. 7A). That quantity equals unity if both pathways provide the same amount of ATP. Furthermore, we determined how well amino acids, fatty acids, lactate and glycerol are suited for the storage of glucose. This measure, termed glucose storage efficiency, is defined as the relative amount of glucose regained if these compounds are produced from glucose and subsequently converted back into glucose (Fig. 7B). Details on the calculation are given in Text S1.

Gluconeogenic energy efficiency and glucose storage efficiencies of a selected list of compounds are displayed in Fig. 6B. We found that 53–74% of the energy remains if fatty acids are used for gluconeogenesis using the most efficient and most inefficient pathways, respectively (pathways 7 and 20). Thus, 26–47% of the energy contained in fatty acids is lost if they are used for gluconeogenesis. For glucogenic amino acids, in contrast, the energy loss is much smaller; the gluconeogenic energy efficiency is in the range from 73% (leucine)–96% (valine) with a value of 87% for the amino acid composition of a typical dietary protein. For glycerol, the gluconeogenic energy efficiency is 95%. These values can explain the particular efficiency of carbohydrate reduced and ketogenic diets like Atkins diet for weight reduction. The reason is likely to be the increased energy loss of gluconeogenesis from fatty acids and ketogenic amino acids in comparison to gluconeogenesis from glucogenic amino acids. Indeed, intermediates of the pathway for gluconeogenesis from fatty acids have been observed in subjects on the Atkins diet [40]. This is also supported by the observation that the traditional diet of inuit does not lead to obesity in spite of the high content in fat. In contrast, new dietary habits of inuit implying a higher consumption in carbohydrates often lead to obesity [48].

Comparing the glucose storage efficiency of the described compounds (Fig. 6B), we find fatty acids, using pathway 7, at the lower end with a storage efficiency of 50% in comparison to 39% for glycine, 78% for alanine and 86% for glycerol. Hence, the conversion of glucose to fatty acids and gluconeogenesis from fatty acids results in a loss of half of the glucose. These values show that fatty acids are not very well suited as glucose storage since their use as such is associated to a higher loss of glucose equivalents of carbohydrates in comparison to glycerol and amino acids that are the major carbohydrate storage compounds besides glycogen. Nevertheless, as discussed above, the utilization of fatty acids as glucose storage gives the body additional flexibility in the utilization of its storage compounds and appears to be used as such in situations during which gluconeogenesis is active. Moreover, both quantities can be useful for examining the effect of caloric restriction on ageing which is known to extend life span in a large number of organisms [49].

### Concluding remarks

Summarizing our findings, it can be concluded that a thorough, systematic and detailed in-silico investigation of the stoichiometrically feasible routes from fatty acids to glucose based on an experimentally corroborated genome-scale metabolic network provides new insight into human metabolism under glucose limitation. It confirms earlier, anecdotal evidence and hypotheses about gluconeogenesis from fatty acids via acetone and provides hitherto unrecognized pathways for that conversion. This provides a plausible explanation for the surprising independence from nutritional carbohydrates over certain periods (e.g. upon the low-carbohydrate diet of inuit, in hibernating animals and embryos of egg-laying animals). Moreover, we provided a detailed analysis of the energetic balance of these pathways, which explains their limited capacity and their contribution to the particular efficiency of carbohydrate reduced and ketogenic diets.

## Materials and Methods

For our analysis we used a genome-scale model of human metabolism [20] in which we split reversible reactions into irreversible forward and backward directions. We set all external metabolites to internal and added an outflow reaction for each of them. We added an inflow reaction for the metabolites NH_4_, Fe^2+^, Fe^3+^, H^+^, K^+^, Ca^2+^, Na^+^, Cl^−^, phosphate, O_2_, and water. To detect pathways converting even-chain fatty acids into glucose, we furthermore added an influx of mitochondrial acetyl-CoA and an outflow of cytosolic glucose 6-phosphate and mitochondrial coenzyme A. Furthermore, we added several reactions of amino acid degradation that were not present in the model and replaced reactions of the respiratory chain by a net reaction since the model could produce ATP without consumption of any other metabolite otherwise. Moreover we constrained the flux through several reactions allowing the unconditional production of ATP to zero (for further details see Text S1). The final network contains 5733 reactions and 3188 metabolites.

### Elementary flux patterns

Elementary flux patterns have been introduced as a new theoretical tool for the analysis of metabolic pathways in genome-scale metabolic networks [21]. Within a subsystem of metabolism, that is, a set of reactions of interest, elementary flux patterns correspond to basic route through that subsystem that are compatible with steady-state fluxes through the entire network. Thus, every elementary flux pattern is associated to at least one steady-state flux of the entire system and corresponds to the set of reactions used by this steady-state flux in the subsystem. The property of elementarity in the definition of elementary flux pattern requires that no elementary flux pattern can be written as set union of other elementary flux patterns. A formal definition will be provided next.

Given the stoichiometric matrix 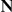 of a reaction network we assume for simplicity that the first 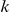 reactions make up the subsystem of interest. A flux pattern 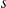 is defined as a set of reactions within the subsystem that is compatible with at least one steady-state flux 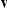 of the entire system. Hence, a set of reaction indices 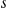 is called a flux pattern if there exists at least one flux vector 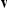 that fulfills the following conditions:
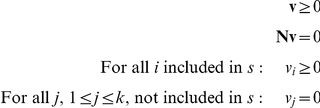



Furthermore, we call a flux pattern elementary if this set of reactions cannot be written as set union, of other flux patterns. For more details see Kaleta *et al* (2009).

### Pathway detection using elementary flux patterns

**Figure 8:**
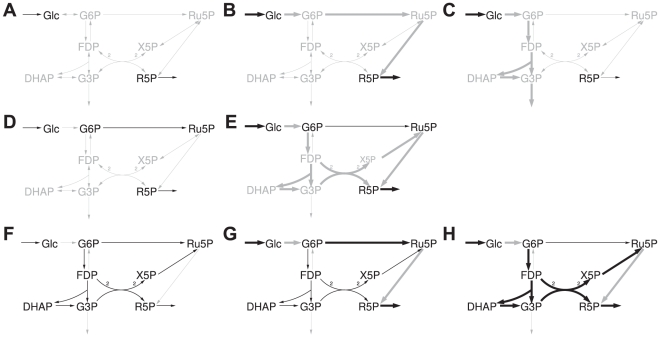
Schematic representation of the iteration process for searching pathways. Black arrows correspond to reactions belonging to the subsystems. **A**, **D** and **F** Subsystems used in the different iteration steps. **B**, **C** and **E** Selected elementary flux patterns (thick black arrows) and the reactions used by an associated elementary mode in the remaining system (thick gray arrows). If only one direction of a reversible reaction belongs to a subsystem, the reverse direction is omitted for clarity. **G** and **H** Elementary flux patterns of the final system producing R5P with the associated pathways through the entire system. A list of abbreviations can be found in Table S1.

Elementary flux patterns can be used to elucidate all possible pathways consuming a certain compound and producing another. This process builds upon a successive expansion of the subsystem under study to reactions that belong to alternative pathways. It will be outlined by way of a small example network comprising glycolysis and the pentose phosphate pathway (Fig. 8). Within this system we want to find all pathways producing ribose-5-phosphate (R5P), a precursor of histidine and nucleotide syntheses from glucose (Glc).

We start with a subsystem encompassing the inflow reaction of Glc and the outflow of R5P (Fig. 8A). Two elementary flux patterns are found. One of them only contains the inflow reaction of Glc and the other the inflow of Glc as well as the outflow of R5P. The first elementary flux pattern indicates the existence of a pathway consuming Glc at steady state without using the outflow of R5P. This corresponds to the glycolytic pathway producing glycerol-3-phosphate (G3P) which is subsequently drained from the system (Fig. 8C). The other flux pattern corresponds to a pathway producing R5P (Fig. 8B). Since we found no elementary flux pattern containing the outflow of R5P without the inflow of Glc we can conclude that the inflow reaction is required for the production of R5P. Otherwise we would have obtained a second elementary flux pattern containing only the outflow of R5P.

Next, we need to determine an elementary mode through the entire system using exactly the reactions of the elementary flux pattern in the subsystem. Such an elementary mode can be obtained using linear programming [21]. This elementary mode corresponds to a first pathway for the production of R5P from Glc. From this initial pathway it is possible to deduce reactions that are essential for the conversion of R5P to Glc (see Text S1 for more details). Thus, we find that in addition to the inflow of Glc, the conversion of Glc to glucose-6-phosphate (G6P) and the conversion of ribulose-5-phosphate (Ru5P) to R5P are required for production of R5P at steady state. The knowledge of essential reactions can simplify the analysis in two ways. First, we do not need to include essential reactions into the subsequent subsystems since every pathway producing R5P will use them anyway. Second, if we find several sequences of essential reactions the task of searching for pathways can be split into sub-tasks. Each sub-task then consists in the search for a pathway connecting a product of a sequence of essential reactions and the educt of the next sequence of essential reactions.

In order to determine reactions that belong to alternative pathways, we include the reactions of the first detected pathway into the subsystem of the next step. As noted above, we need not to add essential reactions and consequently, the subsystem of the second step comprises three reactions (Fig. 8D): the inflow of glucose, the conversion of G6P to Ru5P and the outflow of R5P. This subsystem gives rise to three elementary flux patterns. One of them contains the inflow of G6P and the outflow of R5P (Fig. 8E). This elementary flux pattern corresponds to a pathway for the production of R5P. This flux pattern is associated to an elementary mode that also uses reactions that do not belong to the subsystem (and are not essential reactions). Hence, we have identified reactions that belong to an alternative route. These reactions are subsequently added to the subsystem of the third step (Fig. 8F). In this subsystem we find eight elementary flux patterns, two of which contain the outflow of R5P (Figs. 8G and 8H). Determining the elementary modes associated to these elementary flux patterns, we find that both use only reactions that are either essential for the pathway or belong to the subsystem. Thus, we have identified all pathways producing R5P from Glc. These pathways correspond to the elementary modes associated to each of the two flux patterns containing the outflow of R5P.

### Pathway detection using elementary flux modes

We computed the 100 shortest elementary flux modes producing glucose 6-phosphate from acetyl-CoA using a previously described method [33]. This method allows one to enumerate elementary flux modes with increasing number of reactions. The entire set of elementary flux modes cannot be computed for the genome-scale metabolic network of humans since their number is too large [32]. For the computation we set the metabolites H_2_O, CO_2_, Pi, Ppi, protons, O_2_, ATP, ADP, AMP, GTP, GDP, NADH, NAD^+^, NADPH, NADP^+^, coenzyme A and HCO_3_^+^ to external status to obtain elementary flux modes differing in the underlying carbon conversion pathway rather than in co-factor balancing reactions.

### Calculation of Gibbs free energy changes

We calculated the Gibbs free-energy changes of the described pathways. We performed these calculations for two scenarios: gluconeogenesis from acetyl-CoA and from palmitate. For the first scenario, we assumed that additionally to the energetic balance as depicted in Fig. **6** four moles of acetyl-CoA are converted into one mole of glucose, four moles of coenzyme A and two moles of carbon dioxide. For the second scenario, we assumed that biosynthesis starts from 0.5 moles of palmitate yielding an additional 3.5 moles of NADH as well as FADH and hydrolyzing one mole of ATP through β-oxidation of fatty acids. We calculated the Gibbs free energy change by
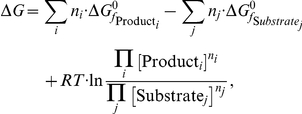
where 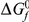 corresponds to the Gibbs free energy of formation, 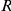 to the universal gas constant, 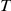 to the temperature, 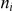 to the stoichiometric coefficient of the 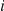
th product and 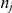 to the stoichiometric coefficient of the 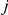
th substrate of the pathway. We assumed a pH-value of 7.2 and a temperature of 37°C. We used measured Gibbs free-energy of formation when available [50], [51] and estimated values otherwise [52]. For concentrations we used measured values if they were available. The corresponding values and references are given in Text S1.

## Supporting Information

Table S1 List of metabolite and enzyme abbreviations.
(PDF)

Text S1 Supplementary material and methods.
(PDF)
