# Supplementary material for: In Silico Evidence for Gluconeogenesis from Fatty Acids in Humans
Source: PLoS Comput Biol. 2011 Jul 21;7(7):e1002116. doi: 10.1371/journal.pcbi.1002116 (PMC3140964; doi:10.1371/journal.pcbi.1002116)
Supplement: Table S1 — List of metabolite and enzyme abbreviations. (PDF) [file pcbi.1002116.s001.pdf]

Table S1: List of abbreviations

List of reactions

Reaction	Catalyzing enzymes
Acat1	acetyl-Coenzyme A acetyltransferase 1 (acetoacetyl Coenzyme A thiolase)
Acat2	acetyl-Coenzyme A acetyltransferase 2 (acetoacetyl Coenzyme A thiolase)
Aco1	aconitase 1, soluble
Aco2	aconitase 2, mitochondrial
Adh	alcohol dehydrogenase IV<br>alcohol dehydrogenase V<br>alcohol dehydrogenase 1A (class I), alpha polypeptide<br>alcohol dehydrogenase IB (class I), beta polypeptide<br>alcohol dehydrogenase 1C (class I), gamma polypeptide<br>alcohol dehydrogenase 6 (class V)<br>alcohol dehydrogenase 7 (class IV), mu or sigma polypeptide<br>alcohol dehydrogenase, iron containing, 1 (liver)<br>alcohol dehydrogenase 1AB<br>alcohol dehydrogenase 1AC
Adh (con't)	alcohol dehydrogenase 1BC
Adh5	alcohol dehydrogenase V
Akr	aldo-keto reductase family 1, member A1<br>aldo-keto reductase family 1, member B1 (aldose reductase)<br>aldo-keto reductase family 7, member A2 (aflatoxin aldehyde reductase)
Akr1b1	aldo-keto reductase family 1, member B1 (aldose reductase)
Akr7a2	aldo-keto reductase family 7, member A2 (aflatoxin aldehyde reductase)
Aldh	aldehyde dehydrogenase 1 family, member A1<br>aldehyde dehydrogenase 1 family, member A2<br>aldehyde dehydrogenase 1 family, member A3<br>aldehyde dehydrogenase 3 family, member A1<br>aldehyde dehydrogenase 3 family, member A2<br>aldehyde dehydrogenase 3 family, member B1<br>aldehyde dehydrogenase 3 family, member B2<br>aldehyde dehydrogenase 7 family, member A1<br>aldehyde dehydrogenase 9 family, member A1
Aqp9	aquaporin 9
Bpgm	2,3-bisphosphoglycerate mutase
Crns<br>(Carnitine shuttle)	Solute carrier family 25, carnitine/acylcarnitine translocase, member 20, mitochondrial<br>carnitine acetyltransferase, mitochondrial
Cyp2e1	cytochrome P450, family 2, subfamily E, polypeptide 1

Reaction	Catalyzing enzymes
Fbp	fructose-1,6-bisphosphatase 1 (liver)<br>fructose-1,6-bisphosphatase 2 (muscle)
Flj32499	hypothetical protein FLJ32499, mitochondrial
Flj39207	C219-reactive peptide
Glo1	glyoxalase I
Grhpr	glyoxylate reductase/hydroxypyruvate reductase
Hadha	hydroxyacyl-Coenzyme A dehydrogenase/3-ketoacyl-Coenzyme A thiolase/enoyl-Coenzyme A hydratase (trifunctional protein), alpha subunit<br>hydroxyacyl-Coenzyme A dehydrogenase/3-ketoacyl-Coenzyme A thiolase/enoyl-Coenzyme A hydratase (trifunctional protein), beta subunit
Hagh	hydroxyacylglutathione hydrolase<br>hydroxyacylglutathione hydrolase-like
Hmgcl	3-hydroxylmethyl-3-methylglutaryl-Coenzyme A lyase<br>3-hydroxymethyl-3-methylglutaryl-Coenzyme A lyase-like 1, mitochondrial
Hmgcs1	3-hydroxy-3-methylglutaryl-Coenzyme A synthase 1 (soluble)
Hmgcs2	3-hydroxy-3-methylglutaryl-Coenzyme A synthase 2 (mitochondrial)
Idh2	isocitrate dehydrogenase 2 (NADP+), mitochondrial
Idh1	isocitrate dehydrogenase 1 (NADP+), soluble
Ireb2	iron-responsive element binding protein
Ldh	lactate dehydrogenase A (liver, skeletal muscle)<br>lactate dehydrogenase B (heart, red blood cell)<br>lactate dehydrogenase C (testis)<br>lactate dehydrogenase A-like 6B (testis)<br>lactate dehydrogenase A-like 6A<br>lactate dehydrogenase (heart, red blood cell, brain, kidney, liver, skeletal muscle)
Ldha	lactate dehydrogenase A (liver, skeletal muscle)
Ldhd	lactate dehydrogenase D
Mdh1	malate dehydrogenase 1 (NAD), soluble<br>malate dehydrogenase 1b (NAD), soluble
Me1	malic enzyme 1, NADP(+)-dependent, cytosolic
Oxct	3-oxoacid CoA transferase 1, mitochondrial<br>3-oxoacid CoA transferase 2, mitochondrial
Pc	pyruvate carboxylase, mitochondrial (liver, kidney, intestine)
Pck1	phosphoenolpyruvate carboxykinase 1, soluble (liver, kidney, intestine)
Pck2	phosphoenolpyruvate carboxykinase 2, mitochondrial (liver, kidney, intestine)
Pgam	brain phosphoglycerate mutase 1<br>muscle phosphoglycerate mutase 2
Pgk	phosphoglycerate kinase 1<br>phosphoglycerate kinase 2 (testis)
Slc16a1	solute carrier family 16 (monocarboxylic acid transporters), member 1

Reaction	Catalyzing enzymes
Slc25a1	solute carrier family 25 (mitochondrial carrier; citrate transporter), member 1
Slc25a10	solute carrier family 25 (dicarboxylate transporter) member 10, mitochondrial
Uev	ubiquitin-conjugating enzyme E2-like
Zadh	zinc binding alcohol dehydrogenase

List of reactions and catalyzing enzymes. In some cases several of the listed proteins are necessary to catalyze the reaction (for more information see the BiGG database, `http://bigg.ucsd.edu`).

Metabolites

Abbreviation	Compound	Abbreviation	Compound
12ppd-S	(S)-propane-1,2-diol	Lac-D	D-lactate
AaCoA	acetoacetyl-CoA	Lac-L	L-lactate
AcAc	acetoacetate	Lald-D	D-lactaldehyde
AcCoA	acetyl-CoA	Lald-L	L-lactaldehyde
CoA	coenzyme A	Lgt-S	(R)-S-lactoylglutathione
DHAP	dihydroxyacetone phosphate	Mal	Malate
FDP	fructose-1,6-bisphosphate	Mthgxl	methylglyoxal
FicytC	ferricytochrome c	OAA	Oxaloacetate
FocytC	ferrocytochrome c	OG	Oxoglutarate
G3P	Glyceraldehyde 3-phosphate	Pyr	pyruvate
G6P	glucose-6 phosphate	R5P	ribose-5-phosphate
Glc	glucose	Ru5P	ribulose-5-phosphate
Gthrd	reduced glutathione	Succ	succinate
HmgCoA	Hydroxymethylglutaryl-CoA	SuccCoA	Succinyl-CoA
Icit	Isocitrate	X5P	xylulose-5-phosphate
