# Supplementary material for: In Silico Evidence for Gluconeogenesis from Fatty Acids in Humans
Source: PLoS Comput Biol. 2011 Jul 21;7(7):e1002116. doi: 10.1371/journal.pcbi.1002116 (PMC3140964; doi:10.1371/journal.pcbi.1002116)
Supplement: Text S1 — Supplementary material and methods. (PDF) [file pcbi.1002116.s002.pdf]

Text S1: Supplementary materials and methods

Elementary flux patterns in ketogenesis

#	Reactions	Enzyme	#	Reactions	Enzyme
1,2	2 AcCoA$_c$ $\rightarrow$ AaCoA$_c$ + CoA$_c$	Acat2	4,5	2 AcCoA$_c$ $\rightarrow$ AaCoA$_c$ + CoA$_c$	Acat2
	AaCoA$_c$ + AcCoA$_c$ $\rightarrow$ HmgCoA$_c$	Hmgcs1		AaCoA$_c$ + AcCoA$_c$ $\rightarrow$ HmgCoA$_c$	Hmgcs1
	HmgCoA$_c$ $\rightarrow$ HmgCoA$_x$	-		HmgCoA$_c$ $\rightarrow$ HmgCoA$_m$	-
	HmgCoA$_x$ $\rightarrow$ Acac$_x$ + AcCoA$_x$	Hmgcl		HmgCoA$_m$ $\rightarrow$ Acac$_m$ + AcCoA$_m$	Hmgcl
	Acac$_x$ $\rightarrow$ Acac$_c$		6	2 AcCoA$_m$ $\rightarrow$ AaCoA$_m$	Acat1
	Acac$_c$ $\rightarrow$ Acac$_m$	Slc16a1		AaCoA$_m$ + Succ$_m$ $\rightarrow$ Acac$_m$ + SuccCoA$_m$	Oxct1
3	2 AcCoA$_m$ $\rightarrow$ AaCoA$_m$ + CoA$_m$	Acat1	6	2 AcCoA$_m$ $\rightarrow$ AaCoA$_m$ + CoA$_m$	Acat1
	AaCoA$_m$ + AcCoA$_m$ $\rightarrow$ HmgCoA$_m$	Hmggcs2		AaCoA$_m$ + AcCoA$_m$ $\rightarrow$ HmgCoA$_m$	Hmggcs2
	HmgCoA$_m$ $\rightarrow$ HmgCoA$_c$	-		HmgCoA$_m$ $\rightarrow$ Acac$_m$ + AcCoA$_m$	Hmgcl
	HmgCoA$_c$ $\rightarrow$ HmgCoA$_x$	-	8,9	2 AcCoA$_c$ $\rightarrow$ AaCoA$_c$ + CoA$_c$	Acat2
	HmgCoA$_x$ $\rightarrow$ Acac$_x$ + AcCoA$_x$	Hmgcl		AaCoA$_c$ $\rightarrow$ Acac$_c$ + CoA$_c$	Aacs
	Acac$_x$ $\rightarrow$ Acac$_c$	-		Acac$_c$ $\rightarrow$ Acac$_m$	Slc16a1
	Acac$_c$ $\rightarrow$ Acac$_m$	Slc16a1			

Table 1: List of pathways for the conversion of acetyl-CoA into acetoacetate. The second column indicates the reactions and the third column the catalyzing enzymes. Note that the pathways 1 an2, 4 and 5, as well as 8 and 9 are identical in reactions and NADPH/NADH-ratio but use a different enzyme for the condensation of two acetyl-CoA to acetoacetyl-CoA. Subscript indices indicate the localization of the metabolites: c - cytosol, m - mitochondrion, x - peroxisom. A list of abbreviations can be found in Table S1.

Elementary flux patterns in acetone degradation

#	Reactions	Enzyme	Rat.	Occ.	#	Reactions	Enzyme	Rat.	Occ.
1	NADPH + Acetol$_c$ $\rightarrow$ NADP$^+$ + 12ppd-S$_c$<br>NAD$^+$ + 12ppd-S$_c$ $\rightarrow$ NADH + Lald-L$_c$<br>NAD$^+$ + Lald-L$_c$ $\rightarrow$ NADH + Lac-L$_c$<br>Lac-L$_c$ $\rightarrow$ Lac-L$_m$<br>NAD$^+$ + Lac-L$_m$ $\rightarrow$ NADH + Pyr$_m$	Akr<br>Add/Zadh<br>Aldh<br>Aqp9<br>Ldha	+2/-3	4	2	NADPH + Acetol$_c$ $\rightarrow$ NADP$^+$ + 12ppd-S$_c$<br>NAD$^+$ + 12ppd-S$_c$ $\rightarrow$ NADH + Lald-L$_c$<br>NAD$^+$ + Lald-L$_c$ $\rightarrow$ NADH + Lac-L$_c$<br>Lac-L$_c$ $\rightarrow$ Lac-L$_m$<br>NAD$^+$ + Lac-L$_m$ $\rightarrow$ NADH + Pyr$_m$	Akr<br>Add/Zadh<br>Aldh<br>Slc16a1<br>Ldha	+2/-3	4
3	NADPH + Acetol$_c$ $\rightarrow$ NADP$^+$ + 12ppd-S$_c$<br>NAD$^+$ + 12ppd-S$_c$ $\rightarrow$ NADH + Lald-L$_c$<br>NAD$^+$ + Lald-L$_c$ $\rightarrow$ NADH + Lac-L$_c$<br>FicytC$_m$ + Lac-L$_c$ $\rightarrow$ FocytC$_m$ + Pyr$_c$<br>Pyr$_c$ $\rightarrow$ Pyr$_m$	Akr<br>Add/Zadh<br>Aldh<br>Flj32499<br>Slc16a1	+2/-3	4	0	NADPH + Acetol$_c$ $\rightarrow$ NADP$^+$ + 12ppd-S$_c$<br>NAD$^+$ + 12ppd-S$_c$ $\rightarrow$ NADH + Lald-L$_c$<br>NAD$^+$ + Lald-L$_c$ $\rightarrow$ NADH + Lac-L$_c$<br>NAD$^+$ + Lac-L$_c$ $\rightarrow$ NADH + Pyr$_c$<br>Pyr$_c$ $\rightarrow$ Pyr$_m$	Akr<br>Add/Zadh<br>Aldh<br>Ldh/Uev3<br>Slc16a1	+2/-3	4
5<br>+<br>6	NADPH + Acetol$_c$ $\rightarrow$ NADP$^+$ + 12ppd-S$_c$<br>NAD$^+$ + 12ppd-S$_c$ $\rightarrow$ NADH + Lald-L$_c$<br>NAD$^+$ + Lald-L$_c$ $\rightarrow$ NADH + Mthglx$_c$<br>NADPH + Mthglx$_c$ $\rightarrow$ NADP$^+$ + Lald-D$_c$<br>NAD$^+$ + Lald-D$_c$ $\rightarrow$ NADH + Lac-D$_c$<br>NAD$^+$ + Lac-D$_c$ $\rightarrow$ NADH + Pyr$_c$<br>Pyr$_c$ $\rightarrow$ Pyr$_m$	Akr<br>Add/Zadh<br>Add/Zadh<br>Akr/Grhpr<br>Aldh<br>Ldhd<br>Slc16a1	+3/-4	0	7	NADPH + Acetol$_c$ $\rightarrow$ NADP$^+$ + 12ppd-S$_c$<br>NAD$^+$ + 12ppd-S$_c$ $\rightarrow$ NADH + Lald-L$_c$<br>NAD$^+$ + Lald-L$_c$ $\rightarrow$ NADH + Mthglx$_c$<br>NADP$^+$ + Mthglx$_c$ $\rightarrow$ NADPH + Pyr$_c$<br>Pyr$_c$ $\rightarrow$ Pyr$_m$	Akr<br>Add/Zadh<br>Add/Zadh<br>Akr1b1<br>Slc16a1	+1/-2	4
8<br>+<br>9	NADPH + Acetol$_c$ $\rightarrow$ NADP$^+$ + 12ppd-S$_c$<br>NAD$^+$ + 12ppd-S$_c$ $\rightarrow$ NADH + Lald-L$_c$<br>NAD$^+$ + Lald-L$_c$ $\rightarrow$ NADH + Mthglx$_c$<br>NADPH + Mthglx$_c$ $\rightarrow$ NADP$^+$ + Lald-D$_c$<br>NAD$^+$ + Lald-D$_c$ + Gthrd$_c$ $\rightarrow$ NADH + Lgt-S$_c$<br>Lgt-S$_c$ $\rightarrow$ Lac-D$_c$ + Gthrd$_c$<br>NAD$^+$ + Lac-D$_c$ $\rightarrow$ NADH + Pyr$_c$<br>Pyr$_c$ $\rightarrow$ Pyr$_m$	Akr<br>Add/Zadh<br>Add/Zadh<br>Akr/Grhpr<br>Adh5<br>Hagh<br>Ldhd<br>Slc16a1	+3/-4	0	10	NADPH + Acetol$_c$ $\rightarrow$ NADP$^+$ + 12ppd-S$_c$<br>NAD$^+$ + 12ppd-S$_c$ $\rightarrow$ NADH + Lald-L$_c$<br>NAD$^+$ + Lald-L$_c$ $\rightarrow$ NADH + Mthglx$_c$<br>NADH + Mthglx$_c$ $\rightarrow$ NAD$^+$ + Lald-D$_c$<br>NAD$^+$ + Lald-D$_c$ $\rightarrow$ NADH + Lac-D$_c$<br>NAD$^+$ + Lac-D$_c$ $\rightarrow$ NADH + Pyr$_c$<br>Pyr$_c$ $\rightarrow$ Pyr$_m$	Akr<br>Add/Zadh<br>Add/Zadh<br>Akr/Grhpr<br>Aldh<br>Ldhd<br>Slc16a1	+2/-3	0
11	NADPH + Acetol$_c$ $\rightarrow$ NADP$^+$ + 12ppd-S$_c$<br>NAD$^+$ + 12ppd-S$_c$ $\rightarrow$ NADH + Lald-L$_c$<br>NAD$^+$ + Lald-L$_c$ $\rightarrow$ NADH + Mthglx$_c$<br>NADH + Mthglx$_c$ $\rightarrow$ NAD$^+$ + Lald-D$_c$<br>NAD$^+$ + Lald-D$_c$ + Gthrd$_c$ $\rightarrow$ NADH + Lgt-S$_c$<br>Lgt-S$_c$ $\rightarrow$ Lac-D$_c$ + Gthrd$_c$<br>NAD$^+$ + Lac-D$_c$ $\rightarrow$ NADH + Pyr$_c$<br>Pyr$_c$ $\rightarrow$ Pyr$_m$	Akr<br>Add/Zadh<br>Add/Zadh<br>Akr/Grhpr<br>Adh5<br>Hagh<br>Ldhd<br>Slc16a1	+2/-3	0	12	NADPH + Acetol$_c$ $\rightarrow$ NADP$^+$ + 12ppd-S$_c$<br>NAD$^+$ + 12ppd-S$_c$ $\rightarrow$ NADH + Lald-L$_c$<br>NAD$^+$ + Lald-L$_c$ $\rightarrow$ NADH + Mthglx$_c$<br>Gthrd$_c$ + Mthglx$_c$ $\rightarrow$ Lgt-S$_c$<br>NADH + Lgt-S$_c$ $\rightarrow$ NAD$^+$ + Lald-D$_c$ + Gthrd$_c$<br>NAD$^+$ + Lald-D$_c$ $\rightarrow$ NADH + Lac-D$_c$<br>NAD$^+$ + Lac-D$_c$ $\rightarrow$ NADH + Pyr$_c$<br>Pyr$_c$ $\rightarrow$ Pyr$_m$	Akr<br>Add/Zadh<br>Add/Zadh<br>Glo1<br>Adh5<br>Aldh<br>Ldhd<br>Slc16a1	+2/-3	0
13	NADPH + Acetol$_c$ $\rightarrow$ NADP$^+$ + 12ppd-S$_c$<br>NAD$^+$ + 12ppd-S$_c$ $\rightarrow$ NADH + Lald-L$_c$<br>NAD$^+$ + Lald-L$_c$ $\rightarrow$ NADH + Mthglx$_c$<br>Gthrd$_c$ + Mthglx$_c$ $\rightarrow$ Lgt-S$_c$<br>Lgt-S$_c$ $\rightarrow$ Lac-D$_c$ + Gthrd$_c$<br>NAD$^+$ + Lac-D$_c$ $\rightarrow$ NADH + Pyr$_c$<br>Pyr$_c$ $\rightarrow$ Pyr$_m$	Akr<br>Add/Zadh<br>Add/Zadh<br>Glo1<br>Hagh<br>Ldhd<br>Slc16a1	+2/-3	0	14	NADPH + Acetol$_c$ $\rightarrow$ NADP$^+$ + Mthglx$_c$<br>NADP$^+$ + Mthglx$_c$ $\rightarrow$ NADPH + Pyr$_c$<br>Pyr$_c$ $\rightarrow$ Pyr$_m$	Cyp2e1<br>Akr1b1<br>Slc16a1	+1/0	69
15<br>+<br>16	NADPH + Acetol$_c$ $\rightarrow$ NADP$^+$ + Mthglx$_c$<br>NADPH + Mthglx$_c$ $\rightarrow$ NADP$^+$ + Lald-D$_c$<br>NAD$^+$ + Lald-D$_c$ $\rightarrow$ NADH + Lac-D$_c$<br>NAD$^+$ + Lac-D$_c$ $\rightarrow$ NADH + Pyr$_c$<br>Pyr$_c$ $\rightarrow$ Pyr$_m$	Cyp2e1<br>Akr/Grhpr<br>Aldh<br>Ldhd<br>Slc16a1	+3/-2	8	17	NADPH + Acetol$_c$ $\rightarrow$ NADP$^+$ + Mthglx$_c$<br>NADH + Mthglx$_c$ $\rightarrow$ NAD$^+$ + Lald-D$_c$<br>NAD$^+$ + Lald-D$_c$ $\rightarrow$ NADH + Lac-D$_c$<br>NAD$^+$ + Lac-D$_c$ $\rightarrow$ NADH + Pyr$_c$<br>Pyr$_c$ $\rightarrow$ Pyr$_m$	Cyp2e1<br>Akr/Grhpr<br>Aldh<br>Ldhd<br>Slc16a1	+2/-1	3
18	NADPH + Acetol$_c$ $\rightarrow$ NADP$^+$ + Mthglx$_c$<br>NADH + Mthglx$_c$ $\rightarrow$ NAD$^+$ + Lald-D$_c$<br>NAD$^+$ + Lald-D$_c$ + Gthrd$_c$ $\rightarrow$ NADH + Lgt-S$_c$<br>Lgt-S$_c$ $\rightarrow$ Lac-D$_c$ + Gthrd$_c$<br>NAD$^+$ + Lac-D$_c$ $\rightarrow$ NADH + Pyr$_c$<br>Pyr$_c$ $\rightarrow$ Pyr$_m$	Cyp2e1<br>Akr/Grhpr<br>Adh5<br>Hagh<br>Ldhd<br>Slc16a1	+2/-1	0	19<br>+<br>20	NADPH + Acetol$_c$ $\rightarrow$ NADP$^+$ + Mthglx$_c$<br>NADPH + Mthglx$_c$ $\rightarrow$ NADP$^+$ + Lald-D$_c$<br>NAD$^+$ + Lald-D$_c$ + Gthrd$_c$ $\rightarrow$ NADH + Lgt-S$_c$<br>Lgt-S$_c$ $\rightarrow$ Lac-D$_c$ + Gthrd$_c$<br>NAD$^+$ + Lac-D$_c$ $\rightarrow$ NADH + Pyr$_c$<br>Pyr$_c$ $\rightarrow$ Pyr$_m$	Cyp2e1<br>Akr/Grhpr<br>Adh5<br>Hagh<br>Ldhd<br>Slc16a1	+3/-2	0
21	NADPH + Acetol$_c$ $\rightarrow$ NADP$^+$ + Mthglx$_c$<br>Gthrd$_c$ + Mthglx$_c$ $\rightarrow$ Lgt-S$_c$<br>NADH + Lgt-S$_c$ $\rightarrow$ NAD$^+$ + Lald-D$_c$ + Gthrd$_c$<br>NAD$^+$ + Lald-D$_c$ $\rightarrow$ NADH + Lac-D$_c$<br>NAD$^+$ + Lac-D$_c$ $\rightarrow$ NADH + Pyr$_c$<br>Pyr$_c$ $\rightarrow$ Pyr$_m$	Cyp2e1<br>Glo1<br>Adh5<br>Aldh<br>Ldhd<br>Slc16a1	+2/-1	0	22	NADPH + Acetol$_c$ $\rightarrow$ NADP$^+$ + Mthglx$_c$<br>Gthrd$_c$ + Mthglx$_c$ $\rightarrow$ Lgt-S$_c$<br>Lgt-S$_c$ $\rightarrow$ Lac-D$_c$ + Gthrd$_c$<br>NAD$^+$ + Lac-D$_c$ $\rightarrow$ NADH + Pyr$_c$<br>Pyr$_c$ $\rightarrow$ Pyr$_m$	Cyp2e1<br>Glo1<br>Hagh<br>Ldhd<br>Slc16a1	+2/-1	4

Table 2: List of pathways for the conversion of acetol to pyruvate. The second column indicates the reactions and the third column the catalyzing enzymes. The forth column gives the ratio of NADPH oxidized to NADH oxidized in each pathway (including the NADPH oxidation for the reduction of acetone to acetol). The fifth column indicates the number of occurrences of the corresponding pathway within the 100-shortest EFMs producing glucose from acetyl-CoA. Ferrocytochrome (FocytC) is considered energetically equivalent to NADH. Pathways shaded in gray have not been reported previously in acetone metabolism (using [1] as a reference). Note that the pathways 5 and 6, 8 and 9, 15 and 16, as well as 19 and 20 are identical in reactions and NADPH/NADH-ratio but use a different enzyme for the reaction of Mthgxl to Lald-D. Except pathways 1 and 2, a direct import is assumed the principal source of mitochondrial pyruvate. Subscript indices indicate the localization of the metabolites: c - cytosol, m - mitochondrion. A list of the abbreviations can be found in Table S1.

Model modifications

Besides the modifications described in the main document we added several reactions to the network. These reactions concern the degradation of propionyl-CoA into succinyl-CoA which are known to occur in humans [2, 3, 4], but were not present in the reconstruction. They are required for the degradation of methionine as well as threonine. The first reaction, catalyzed by propionyl-CoA carboxylase, converts propionyl-CoA to S-methylmalonyl-CoA. This reaction was present in the model, but only in the reverse direction. Thus, we added the following reaction (in the notation of the model)

$$ppcoa + CO_2 \quad \rightarrow \quad H^+ + mmcoa\_S\_c$$

In two subsequent steps, catalyzed by methylmanoyl-CoA epimerase and methylmanoyl-CoA mutase, S-methylmalonyl-CoA is converted into succinyl-CoA. Thus, we added an additional reaction of the form

$$mmcoa\_S\_c \quad \rightarrow \quad succoa$$

Moreover, we found that there exist several sets of reactions in the model that allow the unconstrained conversion of ADP and Pi to ATP without consumption of any energy source. These are mainly transport reactions and the ATP synthase. We remedied this problem by blocking the reactions with the IDs (f-forward reaction blocked, b-backward direction blocked) "R_BILGLCURte"(b), "R_BILDGLCURte"(b), "R_ACOAO7p"(f), "R_RDH1a"(f) and "R_ATPS4m"(f). The last reaction corresponds to ATP synthase, the final step in the respiratory chain. Thus, we added two reactions to represent the respiratory chain (all metabolites in the mitochondrial form):

$$\begin{aligned} NADH + H^+ + 2.5\ ADP + 2.5\ Pi + 0.5\ O_2 &\rightarrow NAD + 2.5\ ATP + 3.5\ H_2O \\ FADH2 + 1.5\ ADP + 1.5\ Pi + 0.5\ O_2 &\rightarrow FAD + 1.5\ ATP + 2.5\ H_2O \end{aligned}$$

Determining reactions essential for a pathway

In order to determine which reactions are essential on a pathway a linear program can be used. It consists in checking whether there still exists a steady-state flux through the final reaction of the pathway, if the flux through each reaction of an initial pathway is individually constrained to zero. If we find no steady-state flux, the corresponding reaction is essential for the pathway.

We use the same notation like in the main document. Thus, $\mathbf{N}$ corresponds to the $m \times n$ stoichiometric matrix of a reaction network of m metabolites and n reactions. Given an initial pathway of length l, that is, a set of indices of reactions, $i_1, ..., i_l$ with $1 \leq i_1, ..., i_l \leq n$ we assume that i_l is the index of the final reaction r_{i_l} of the pathway. In order to determine the essential reactions of the pathway we check for each i_p with $1 \leq p \leq l - 1$ whether the linear program

$$\mathbf{N} \cdot \mathbf{v} = 0 \tag{1}$$

$$\mathbf{v} \geq 0 \tag{2}$$

$$v_{i_p} = 0 \tag{3}$$

$$v_{i_l} \geq 1 \tag{4}$$

with the variables $\mathbf{v}$ is feasible. Thus, we check if there still exists a steady-state flux through r_{i_l} if constraining the flux through r_{i_p}, v_{i_p}, to zero. In consequence, if the linear program in infeasible for a reaction i_p, r_{i_p} is an essential reaction. Note that we split reversible reactions into irreversible forward and backward direction. Hence, equation (2) corresponds to an irreversibility constraint.

ATP consumption for gluconeogenesis from fatty acids

Here we want to discuss the requirements in terms of NADPH, NADH and ATP of the presented pathways. A similar analysis of energetic requirements in acetone metabolism can be found in [5]. In the following, when speaking of quantities of metabolites, we will refer to moles of each metabolite without specifically stating this. Overall, one glucose and two carbon dioxide are produced from four acetyl-CoA. The following considerations will focus on the production of one glyceraldehyde 3-phosphate from two acetyl-CoA. The following steps in gluconeogenesis produce glucose from two glyceraldehyde 3-phosphate without further NADH or ATP consumption. It is assumed that a gradient of ten protons from intermembrane space to the inner mitochondrion equals an amount of 2.5 ATP that can be produced from oxidation of one mitochondrial NADH [6].

The alternative pathways in ketone metabolism only yield energy in form of ATP or GTP, if acetoacetate is produced from acetoacetyl-CoA. In one case, via the 3-Oxoacid CoA transferase, one succinyl-CoA is produced from succinate and coenzyme A. Succinyl-CoA can be hydrolyzed via production of one ATP or one GTP. In the cytosol, acetoacetate can be produced directly from acetoacetyl-CoA accompanied with production of one ATP from AMP and pyrophosphate. When equaling the phosphorylation of AMP to ATP with two times the reaction of ADP to ATP, the production of acetoacetate can yield in between zero and two ATP. The primary site of production of ketone bodies is the mitochondrial matrix. Hence, we base our calculations on the pathway via 3-Oxoacid CoA transferase since it produces one ATP in contrast to the alternative pathway via the intermediate of hydroxymethylglutaryl-CoA in which no ATP is being produced.

In acetone metabolism there are large differences in terms of NADPH oxidized and NAD^+reduced in the different pathways. The reduction of one ferricytochrome can be equaled with the reduction of one NAD^+. Replenishing one NADPH using the described pathway via the malic enzyme costs an equivalent of $3\frac{1}{2}$ ATP (Fig. 5 in main document). One ATP is consumed in the carboxylation of pyruvate to oxaloacetate. The reduction of oxaloacetate to malate oxidizes one NADH. Consequently, the import of pyruvate and carboxylation to oxaloacetate costs an equivalent of one ATP and the reduction to malate with subsequent transport into the cytosol 2.5 ATP.

Further down in the pathway, the conversion of one cytosolic pyruvate to cytosolic phosphoenolpyruvate costs $2\frac{1}{2}$ ATP. This is the cost of the cycling pathway described above plus the hydrolization of one GTP to decarboxylate oxaloacetate. In the following, one ATP and one NADH are necessary to form glyceraldehyde 3-phosphate. This sums up to a cost of 3 ATP.

Overall the energy requirements of the pathways are mainly determined by the pathway used in ketogenesis and acetone metabolism. The most efficient pathway in acetone metabolism is pathway 7 (Table) which has a netto-balance of one NADPH consumed and 2 NADH produced. Overall this would result in an investment of

$-$	1 ATP	(conversion of acetyl-CoA to acetone)
$+$	$(1 \times 3\frac{1}{2})$ ATP	(NADPH replenishment)
$-$	(2×2.5) ATP	(cytosolic NADH in acetone metabolism)
$+$	$5\frac{1}{2}$ ATP	(Pyruvate to glyceralde 3-phosphate)
$+$	**3 ATP**	(overall ATP consumption)

Thus, the production of one glucose from 4 acetyl-CoA costs at least 6 ATP. Using palmitic acid as starting compound instead we additionally need to take into account ATP produced during β-oxidation of palmitic acid. Starting from 0.5 palmitic acid, 4 acetyl-CoA can be produced and 13 ADP are converted into ATP [6]. Thus, there is a net production of $13 - 6 = 7$ ATP if producing one glucose from 0.5 palmitic acid.

Taking by contrast a pathway which is in favor of NADPH consumption over NADH production, the

balance is (pathway 14, Table)

$-$	1 ATP	(conversion of acetyl-CoA to acetone)
$+$	$(1 \times 3\frac{1}{2})$	(NADPH replenishment)
$-$	(0×2.5) ATP	(cytosolic NADH in acetone metabolism)
$+$	$5\frac{1}{2}$ ATP	(Pyruvate to glyceralde 3-phosphate)
$+$	**8 ATP**	(overall ATP consumption)

resulting in a cost of 16 ATP for each glucose produced. Starting from palmitic acid, each glucose costs $16 - 13 = 3$ ATP.

The most ATP-intensive routes can be constructed from pathways 15, 16, 19 or 20 and no ATP production in ketogenesis

$-$	0 ATP	(conversion of acetyl-CoA to acetone)
$+$	$(3 \times 3\frac{1}{2}$ ATP)	(NADPH replenishment)
$-$	(2×2.5) ATP	(cytosolic NADH in acetone metabolism)
$+$	$5\frac{1}{2}$ ATP	(Pyruvate to glyceralde 3-phosphate)
$+$	**11 ATP**	(overall ATP consumption)

This requires 22 ATP to produce one glucose. Starting from palmitic acid, each glucose costs $22 - 13 = 9$ ATP.

Computation of storage efficiencies

Computation of gluconeogenic energy efficiencies

The gluconeogenic energy efficiency relates the amount of ATP that can be produced by directly oxidizing a compound c to the amount of ATP that can be obtained by oxidizing the amount of glucose that can be produced from c. For the computations we added an inflow reaction of c to the metabolic network used in the main manuscript:

$$\rightarrow c.$$

Additionally, we added a reaction of the form:

$$1\ ATP\ +\ 1\ H_2O \rightarrow 1\ ADP\ +\ 1\ Pi +\ 1\ H^+.$$

that mimics ATP hydrolyzing reactions. Then we computed the gluconeogenic energy efficiency $e_{energy}(c)$ of a compound c as follows.

First, we computed the maximum amount of ATP that can be produced from completely oxidizing c, $max_{ATP}^{(1)}(c)$. This we did by optimizing the following linear program where $\mathbf{N^{(1)}}$ represents the stoichiometric matrix with the two previously mentioned reactions added (v_{inflow_c} – flux through inflow reaction of c, v_{ATP} – flux through ATP hydrolysis reaction):

$$max\ v_{ATP}$$

s.t.

$$\begin{aligned} \mathbf{N^{(1)}} \cdot \mathbf{v} &= 0 \\ \mathbf{v} &\geq 0 \\ v_{inflow_c} &\leq 1 \end{aligned}$$

The optimal value of the objective function corresponds to the maximum amount of ATP, $max_{ATP}^{(1)}(c)$, that can be produced from oxidizing c. Please note that this includes cases in which c cannot be completely oxidized by the metabolic network but some derivative of c is excreted.

Second, we computed the maximum amount of glucose that can be produced from each compound c, $max_{Glc}(c)$, by adding an outflow reaction of glucose

$$Glucose \rightarrow$$

and solving the following linear program in which $\mathbf{N^{(2)}}$ corresponds to $\mathbf{N^{(1)}}$ with the outflow of glucose added (($v_{outflow_{Glc}}$ – flux through outflow reaction of glucose):

$$max\ v_{Glc}$$

s.t.

$$\begin{aligned} \mathbf{N^{(2)}} \cdot \mathbf{v} &= 0 \\ \mathbf{v} &\geq 0 \\ v_{inflow_c} &\leq 1 \end{aligned}$$

The optimal value of the objective function corresponds to the maximum amount of glucose, $max_{Glc}(c)$, that can be obtained from c. If gluconeogenesis from a particular compound c is not possible, $max_{Glc}(c)$ is zero.

Third, in some cases gluconeogenesis starting from a compound can yield additional ATP. Thus, we computed the maximum amount of ATP that can be obtained during gluconeogenesis from a particular compound c, assuming a maximum of glucose produced, $max^{(2)}_{ATP}(c)$. This we did by fixing the outflow of glucose to the maximum, $max_{Glc}(c)$, and maximizing the flux through the ATP hydrolysis reaction:

$$max\ v_{ATP}$$

s.t.

$$\begin{aligned} \mathbf{N^{(2)}} \cdot \mathbf{v} &= 0 \\ \mathbf{v} &\geq 0 \\ v_{inflow_c} &\leq 1 \\ v_{Glc} &= max_{Glc}(c) \end{aligned}$$

The optimal value of the objective function corresponds to the maximum amount of ATP, $max^{(2)}_{ATP}(c)$, that can be obtained during gluconeogenesis from c assuming the maximum of glucose produced. If gluconeogenesis from a particular compound c is not possible the linear program is infeasible.

Multiplying $max_{Glc}(c)$ with the 32 ATP that can be produced by completely oxidizing one glucose in humans [6] and adding $max^{(2)}_{ATP}(c)$ we obtain the maximum amount of ATP that can be produced during and after gluconeogenesis from c. Relating this value to the amount of ATP that can be produced by directly oxidizing c we obtain the gluconeogenic energy efficiency $e_{energy}(c)$ by

$$e_{energy}(c) = \frac{max_{Glc}(c) \cdot 32 + max^{(2)}_{ATP}(c)}{max^{(1)}_{ATP}(c)}. \tag{5}$$

Computation of glucose storage efficiencies

The glucose storage efficiency of a particular compound c, $e_{storage}(c)$, relates the amount of glucose that can be produced from one c to the amount of glucose that is required to produce one c. From the last subsection we have already obtained the maximum amount of glucose that can be produced from compound c, $max_{Glc}(c)$. Thus, we additionally require the minimal amount of glucose that is required

for the production of one c, $min_{Glc\_prod\_c}(c)$. This value can be obtained by adding an inflow reaction of glucose

$$\rightarrow Glucose$$

and an outflow reaction of c

$$c \rightarrow$$

to the original metabolic network. Then we optimize the following linear program in which $\mathbf{N^{(3)}}$ corresponds to the initial stoichiometric matrix to which the two above reactions have been added ($v_{inflow_{Glc}}$ – flux through inflow reaction of Glc, $v_{outflow_c}$ – flux through outflow reaction of c):

$$min\ v_{inflow_{Glc}}$$

s.t.

$$\begin{aligned} \mathbf{N^{(3)}} \cdot \mathbf{v} &= 0 \\ \mathbf{v} &\geq 0 \\ v_{outflow_c} &= 1 \end{aligned}$$

The optimal value of the objective function corresponds to the minimal amount of glucose, $min_{Glc\_prod\_c}(c)$, that is required for the production of one c. If the biosynthesis of a particular compound c from glucose is not possible the linear program is infeasible.

Thus, we obtain the glucose storage efficiency of c, $e_{storage}(c)$, by

$$e_{storage}(c) = \frac{max_{Glc}(c)}{min_{Glc\_prod\_c}(c)}. \quad (6)$$

Storage efficiencies in humans and Escherichia coli

We computed gluconeogenic energy efficiencies and glucose storage efficiencies for important storage compounds in humans. We also computed these values for *E. coli*, which possesses a native glyoxylate shunt, for comparison. For *E. coli* we used the genome-scale metabolic model of Feist et al. [7]. For amino acids we modified Eq. 6 by additionally subtracting 4 ATP in the numerator to take into account the storage of amino acids in proteins (cost of amino acid polymerization into proteins). The results are listed in the following table. For palmitic acid we give values for the most efficient pathway (for humans, pathway 7 in Tab.) and the most inefficient pathway (for humans, pathways 15, 16, 19 or 20 in Tab. and no ATP production in ketogenesis). Compounds for which "-" is given lack a degradation pathway in case of the gluconeogenic energy efficiency, and either a biosynthetic or degradation pathway in case of the glucose storage efficiency.

Compound	Humans		*Escherichia coli*	
	e_{energy}	$e_{storage}$	e_{energy}	$e_{storage}$
Alanine	88.9%	77.8%	85.5%	80.0%
Cysteine	88.9%	-	85.5%	34.7%
Aspartate	94.2%	61.8%	92.2%	82.2%
Glutamate	88.4%	68.4%	89.5%	75.9%
Phenylalanine	83.8%	-	-	-
Glycine	84.1%	39.4%	85.5%	55.9%
Histidine	85.4%	-	-	-
Isoleucine	88.4%	-	-	-
Lysine	81.3%	-	-	-
Leucine	73.1%	-	-	-
Methionine	92.3%	-	-	-
Asparagine	94.0%	57.3%	92.2%	82.2%
Proline	84.3%	71.7%	90.3%	59.4%
Glutamine	88.4%	67.0%	89.5%	73.5%
Arginine	85.0%	57.8%	88.3%	64.9%
Serine	84.1%	71.0%	85.5%	67.3%
Threonine	90.5%	-	81.1%	63.8%
Valine	96.4%	-	-	-
Tryptophan	80.2%	-	85.5%	16.3%
Tyrosine	82.9%	-	-	-
Glycerol	94.6%	85.8%	96.3%	80.0%
Lactate	88.9%	83.3%	85.5%	80.0%
Palmitic acid (eff.)	73.6%	50.4%	85.5%	42.8%
Palmitic acid (ineff.)	53.3%	44.6%	n.a.	n.a.
Bovine α-Actin	86.8%	-	-	-

$\triangle$G-values and concentrations of compounds

For the calculation of the ΔG-values of the presented pathways, Gibbs free energies of formation and concentrations of the converted metabolites are required. Where possible, measured Gibbs free energies of formation from [8] as well as [9] and otherwise estimated values from [10] were used. For ATP, ADP and Pi we used the difference of the ΔG-values between the substrates and products of ATP hydrolization instead of the individual values.

As concentrations we used data from rat hepatocyte, the primary site of gluconeogenesis, where possible and a standard concentration of $10^{-3}\,\frac{mol}{l}$ otherwise. There were no principal changes in the resulting ΔG-values when varying this value. Total NADH/NADH+ and NADPH/NADP+ concentrations in rat liver were measured in [11]. The relative fraction of the reduced to the oxidized state for NADH/NADH+ as well as NADPH/NADP+ in mitochondria and cytosol of rat liver were taken from [12].

**Compound**	$\mathbf{\Delta G^0_f}$ ($\frac{kJ}{mol}$)	**Concentration** ($\frac{mol}{l}$)	**Comment**
Acetyl-CoA (mit.)	-3290 [10]	$8.6{\cdot}10^{-4}$ [13]	fasted rat hepatocyte (mitochondrial)
Glucose (cyt.)	-917 [8]	10^{-3}	
CO2 (mit./cyt.)	-394 [8]	10^{-3}	
CoA (mit.)	-3148 [10]	$2.4{\cdot}10^{-3}$ [13]	fasted rat hepatocyte (mitochondrial)
ATP (mit.)	-30.5 [8]	$2.82{\cdot}10^{-3}$ [11]	rat hepatocyte
ATP (cyt.)	-30.5 [8]	$2.82{\cdot}10^{-3}$ [11]	rat hepatocyte
NADPH (cyt.)	-809 [9]	$1.16{\cdot}10^{-4}$ [11, 12]	rat hepatocyte
NADH (cyt.)	22.7 [9]	$1.25{\cdot}10^{-4}$ [11, 12]	rat hepatocyte
Palmitat (mit.)	-305 [8]	10^{-3}	
FADH (mit.)	-2253 [10]	10^{-3}	
NADH (mit.)	22.7 [9]	$3.15{\cdot}10^{-4}$ [11, 12]	rat hepatocyte
NADP (cyt.)	-835 [9]	$3.42{\cdot}10^{-5}$ [11, 12]	rat hepatocyte
NAD (cyt.)	0 [9]	$8.95{\cdot}10^{-4}$ [11, 12]	rat hepatocyte
ADP (cyt.)	0	$6.5{\cdot}10^{-4}$ [11]	rat hepatocyte
Pi (cyt.)	0	10^{-3}	
FAD (mit.)	-2216 [10]	10^{-3}	
ADP (mit.)	0	$6.5{\cdot}10^{-4}$ [11]	rat hepatocyte
Pi (mit.)	0	10^{-3}	
NAD (mit.)	0 [9]	$7.05{\cdot}10^{-4}$ [11, 12]	rat hepatocyte
H (mit./cyt.)	0	$6.31{\cdot}10^{-8}$	cellular pH of 7.2

Table 3: ΔG-values and concentrations of substrates and products of the presented pathways. Sources for values are indicated in brackets. Abbreviations: mit., mitochondrial; cyt., cytosolic.

References

[1] Kalapos MP (2003) On the mammalian acetone metabolism: from chemistry to clinical implications. Biochim Biophys Acta 1621: 122–139.

[2] Jansen R, Kalousek F, Fenton WA, Rosenberg LE, Ledley FD (1989) Cloning of full-length methylmalonyl-coa mutase from a cdna library using the polymerase chain reaction. Genomics 4: 198–205.

[3] Bobik TA, Rasche ME (2001) Identification of the human methylmalonyl-coa racemase gene based on the analysis of prokaryotic gene arrangements. implications for decoding the human genome. J Biol Chem 276: 37194–37198.

[4] Mootha VK, Bunkenborg J, Olsen JV, Hjerrild M, Wisniewski JR, et al. (2003) Integrated analysis of protein composition, tissue diversity, and gene regulation in mouse mitochondria. Cell 115: 629–640.

[5] Kalapos MP (1999) Possible physiological roles of acetone metabolism in humans. Med Hypotheses 53: 236–242.

[6] Lehninger A, Nelson DL, Cox MM (2008) Lehninger Principles of Biochemistry. W. H. Freeman, 5th edition.

[7] Feist AM, Henry CS, Reed JL, Krummenacker M, Joyce AR, et al. (2007) A genome-scale metabolic reconstruction for *Escherichia coli* K-12 MG1655 that accounts for 1260 ORFs and thermodynamic information. Mol Syst Biol 3: 121.

[8] Thauer RK, Jungermann K, Decker K (1977) Energy conservation in chemotrophic anaerobic bacteria. Bacteriol Rev 41: 100–180.

[9] Alberty RA (2004) Thermodynamic properties of nucleotide reductase reactions. Biochemistry 43: 9840–9845.

[10] Jankowski MD, Henry CS, Broadbelt LJ, Hatzimanikatis V (2008) Group contribution method for thermodynamic analysis of complex metabolic networks. Biophys J 95: 1487–1499.

[11] Zydowo MM, Smoleski RT, Swierczyski J (1993) Acetate-induced changes of adenine nucleotide levels in rat liver. Metabolism 42: 644–648.

[12] Williamson DH, Lund P, Krebs HA (1967) The redox state of free nicotinamide-adenine dinucleotide in the cytoplasm and mitochondria of rat liver. Biochem J 103: 514–527.

[13] Siess EA, Brocks DG, Wieland OH (1976) Subcellular distribution of key metabolites in isolated liver cells from fasted rats. FEBS Lett 69: 265–271.
